# A Mixed-Method Study of the Utilization and Determinants of Private Health Insurance Schemes in the Residents of Rural Communities in Central India: A Study Protocol

**DOI:** 10.7759/cureus.63648

**Published:** 2024-07-02

**Authors:** Pankaj C Jambholkar, Sonali G Choudhari, Aditya Dhonde

**Affiliations:** 1 Community Medicine, Jawaharlal Nehru Medical College, Datta Meghe Institute of Higher Education and Research, Wardha, IND

**Keywords:** mixed-method study, determinants, utilization, central india, rural communities, private health insurance

## Abstract

Background

Private health insurance plays a critical role in healthcare financing, yet its utilization and determinants in rural settings still need to be studied, particularly in Central India. This study aims to address this gap by investigating the utilization and determinants of private health insurance schemes among residents of rural communities in Central India.

Materials and methods

A convergent parallel mixed-method study design, consisting of quantitative and qualitative approaches, will be employed. Quantitative data will be collected through structured questionnaires administered to residents aged 18 and above within the field practice area of a tertiary medical college hospital. Qualitative data will be gathered through in-depth interviews with key stakeholders. Statistical analysis will include descriptive and inferential statistics, while thematic analysis will be employed for qualitative data (CTRI Number CTRI/2024/06/069155).

Conclusion

The findings of this study will provide valuable insights into the utilization and determinants of private health insurance schemes in rural communities of Central India. By identifying barriers and facilitators to insurance uptake, policymakers and healthcare providers can develop targeted interventions to improve healthcare access and affordability in rural areas. In addition, the study will contribute to the existing literature on private health insurance utilization in India, informing future research endeavors and policy initiatives.

## Introduction

In recent years, there has been a growing recognition of the importance of private health insurance in complementing public healthcare systems and providing financial protection against the rising costs of medical care [1]. In India, where healthcare expenditures often significantly burden households, private health insurance has emerged as a critical mechanism for accessing healthcare services and managing healthcare-related expenses [2]. However, the utilization of private health insurance needs to be more distributed, with rural populations often facing barriers to access and affordability [3]. Despite efforts to promote insurance coverage, disparities persist, highlighting the need for a deeper understanding of the determinants influencing insurance uptake in rural areas [4]. Factors such as low awareness, limited financial resources, and cultural perceptions may contribute to the underutilization of private health insurance among rural residents [5].

Central India's predominantly rural population and diverse socioeconomic landscape provide an essential context for examining the utilization and determinants of private health insurance schemes [6]. Investigating the awareness levels, utilization patterns, and perceptions of rural communities toward private health insurance aims to generate evidence-based insights that can inform policy decisions and interventions to improve access to healthcare financing in the region. Through a mixed-method approach, combining quantitative surveys and qualitative interviews, this study seeks to comprehensively understand the factors shaping private health insurance utilization in rural Central India. By elucidating the sociodemographic, economic, and cultural determinants of insurance uptake, we aim to contribute to developing targeted strategies for expanding insurance coverage and enhancing financial protection for rural populations.

## Materials and methods

Study design

This study employs a mixed-method design, combining both quantitative and qualitative approaches to comprehensively explore the utilization and determinants of private health insurance schemes in the rural community of Central India. Specifically, it adopts a convergent parallel design, wherein quantitative and qualitative data are collected concurrently and given equal weight in analysis and interpretation.

Participants and eligibility criteria

The study participants are residents of rural areas within the field practice area affiliated with the Department of Community Medicine under Datta Meghe Institute of Higher Education & Research, a tertiary medical college hospital in Central India. To be eligible for participation, individuals must be 18 years or older and willing to participate in the study. Exclusion criteria encompass individuals who are severely ill and incapable of completing the study questionnaire due to their health condition.

Data collection procedure

Quantitative Data Collection

Trained fieldworkers will administer a structured questionnaire via the Kobotool platform to gather quantitative data. The questionnaire is designed to assess various aspects, including awareness of private health insurance schemes, patterns of utilization, and factors influencing uptake. Using systematic random sampling, households within the study area will be selected for participation. Before data collection, informed consent will be obtained from all participants. Data collectors conducted face-to-face interviews, and responses will be recorded directly in the electronic questionnaire. Collected data will be securely stored and transferred to a Microsoft Excel worksheet for subsequent analysis using IBM SPSS Statistics version 29.0.2 (IBM Corp., Armonk, NY).

Qualitative Data Collection

In-depth interviews will be conducted with key stakeholders selected from the same primary health center area. These stakeholders may include influential figures, such as Sarpanch, Gram Panchayat Members, CMS of the tertiary healthcare institution, health insurance officers, and medical social workers. Semi-structured interview guides, prepared in Marathi and validated through pilot testing, will be utilized during these interviews. Approximately 20 to 30 in-depth interviews will be conducted until data saturation is reached, ensuring a comprehensive understanding of the participants' perspectives and experiences. Interviews will be audio-recorded with the participants' consent and transcribed verbatim for qualitative analysis. Thematic analysis will be employed to identify recurring patterns, themes, and insights related to private health insurance schemes from the qualitative data.

Outcomes

The primary objective of this study is to ascertain the extent to which residents of rural communities in Central India utilize private health insurance schemes. By examining the utilization rates, the study aims to provide insights into the current level of coverage and access to private health insurance among rural populations. This primary outcome will shed light on the effectiveness of private health insurance as a means of healthcare financing in rural settings, informing future policy decisions and interventions to improve healthcare access and affordability.

In addition to the primary outcome, several secondary outcomes will be explored. First, the study seeks to assess the level of awareness regarding private health insurance schemes among rural residents. Understanding the extent of knowledge about available insurance options will provide valuable context for interpreting utilization rates and identifying potential barriers to uptake. Furthermore, the study aims to elucidate the factors influencing individuals' decisions to utilize or abstain from private health insurance. By examining these determinants, such as economic status, education level, and perceived value of insurance, the study aims to identify targets for interventions aimed at increasing insurance coverage in rural areas.

Another secondary outcome of interest is the exploration of participants' perceptions and attitudes toward private health insurance. Through qualitative interviews, the study will delve into individuals' beliefs, concerns, and expectations regarding health insurance, offering valuable qualitative insights into the role of insurance in healthcare-seeking behavior. In addition, the study will investigate sociodemographic determinants associated with private health insurance utilization. By analyzing factors such as age, gender, household income, and occupation, the study aims to identify demographic groups that may be particularly vulnerable to underinsurance or lack of coverage.

Statistical analysis

Quantitative data collected through the structured questionnaire will undergo a rigorous statistical analysis. Descriptive statistics, including frequencies, percentages, means, and standard deviations, will be used to summarize the characteristics of the study population and critical variables. These descriptive analyses will provide a comprehensive overview of the demographic profile of participants and their level of engagement with private health insurance. Furthermore, inferential statistics, such as chi-square tests and t-tests, will be employed to explore associations between variables and identify determinants influencing private health insurance uptake. Regression analysis may also examine the relationship between sociodemographic factors and insurance utilization, controlling for potential confounding variables. All statistical analyses will be conducted using SPSS version 29.0.2, with a predetermined significance level set at p < 0.05 to determine statistical significance.

Ethics and dissemination

Ethical considerations are paramount throughout all stages of the study. Approval will be sought from the Ethical Committee of Datta Meghe Institute of Higher Education & Research (approval no. DMIHER(DU)/IEC/2024/132) and the Clinical Trials Registry of India (CTRI) (CTRI no. CTRI/2024/06/069155) to ensure that the study adheres to the highest ethical standards. Informed consent will be obtained from all study participants before data collection, with strict protocols to protect confidentiality and privacy. The dissemination of study findings is essential for maximizing the impact of research efforts. Results will be disseminated through peer-reviewed publications in reputable academic journals and presentations at relevant conferences and seminars. In addition, key findings will be shared with stakeholders, including policymakers, healthcare providers, and community leaders, to inform decision-making and facilitate the development of targeted interventions. Open access to study findings will be encouraged to ensure widespread access and utilization of research outcomes to benefit rural communities and healthcare systems in Central India.

## Results

The results of the study will be drawn after the study is conducted in 2025. We expect a positive impact on the population to utilization and determinants of private health insurance schemes in the residents of rural communities in central India.

## Discussion

The proposed study seeks to investigate the utilization and determinants of private health insurance schemes in the rural communities of Central India. The findings of this study are expected to contribute to the existing literature on healthcare financing and access in rural India. Previous research has highlighted the challenges faced by rural populations in accessing affordable and quality healthcare services, with financial barriers often cited as a significant impediment [7]. Private health insurance has the potential to mitigate some of these barriers by providing financial protection against healthcare costs and facilitating access to healthcare services [8]. However, the extent rural residents utilize private health insurance and the factors influencing uptake still need to be better understood.

The anticipated findings of this study may have several important implications for policy and practice. First, by providing quantitative estimates of private health insurance coverage and awareness levels among rural populations, the study can inform policymakers about the current status of insurance penetration in rural areas. This information is crucial for designing targeted interventions to increase awareness and uptake of private health insurance schemes. For example, community-based education and outreach programs may be implemented to raise awareness about the benefits of health insurance and dispel misconceptions.

The study also aims to identify determinants influencing individuals' decisions to enroll in private health insurance or forego coverage. Sociodemographic factors such as income, education, and occupation are expected to significantly shape the insurance uptake [9]. Understanding these determinants can help policymakers design policies and programs that target vulnerable populations and address barriers to access. For instance, subsidies or incentives may be provided to low-income individuals to encourage enrollment in private health insurance schemes [10].

Furthermore, qualitative insights obtained through in-depth interviews will offer a nuanced understanding of participants' perceptions and attitudes toward private health insurance [11]. These insights can inform the design of culturally appropriate insurance products and communication strategies tailored to the needs and preferences of rural communities. By addressing concerns and misconceptions, policymakers can build trust and confidence in private health insurance schemes, encouraging greater uptake among rural residents.

## Conclusions

This study aims to comprehensively investigate the utilization and determinants of private health insurance schemes in the rural communities of Central India. Through a mixed-method approach combining quantitative surveys and qualitative interviews, the research provides a nuanced understanding of insurance coverage patterns, factors influencing uptake, and individuals' perceptions of insurance. By rigorously analyzing the data collected and interpreting thematic insights, the study seeks to offer valuable evidence for policymakers, healthcare providers, and community leaders to formulate targeted interventions to expand insurance coverage and improve financial protection against healthcare costs in rural areas. Ultimately, by disseminating the study findings widely and engaging stakeholders in meaningful dialogue, this research aspires to advance healthcare access and outcomes for rural populations in Central India, fostering a more equitable and resilient healthcare system for all.
